# Pharmacologic inhibition of PI3K p110δ in mutant Shp2E76K-expressing mice

**DOI:** 10.18632/oncotarget.21455

**Published:** 2017-10-03

**Authors:** Lisa Deng, Elizabeth L. Virts, Reuben Kapur, Rebecca J. Chan

**Affiliations:** ^1^ Herman B Wells Center for Pediatric Research, Indiana University School of Medicine, Indianapolis, IN, USA; ^2^ Department of Medical & Molecular Genetics, Indiana University School of Medicine, Indianapolis, IN, USA; ^3^ Department of Biochemistry & Molecular Biology, Indiana University School of Medicine, Indianapolis, IN, USA; ^4^ Department of Pediatrics, Indiana University School of Medicine, Indianapolis, IN, USA

**Keywords:** JMML, Shp2, PI3K p110δ, mouse model, in vivo

## Abstract

Juvenile myelomonocytic leukemia is a childhood malignancy that lacks effective chemotherapies and thus has poor patient outcomes. PI3K p110δ has been found to promote hyperproliferation of cells expressing mutant Shp2. In this study, we tested the efficacy of a PI3Kδ inhibitor in mice expressing the Shp2 gain-of-function mutation, E76K. We found that *in vivo* treatment of mice led to significantly decreased splenomegaly, reduced frequency of bone marrow progenitor cells, and increased terminally differentiated peripheral blood myeloid cells. The survival of drug-treated mice was significantly prolonged compared to vehicle-treated controls, although mice from both groups ultimately succumbed to a similar myeloid cell expansion. PI3Kδ inhibitors are currently used to treat patients with relapsed lymphoid malignancies, such as chronic lymphocytic leukemia. The current findings provide evidence for using PI3Kδ inhibitors as a treatment strategy for JMML and potentially other myeloid diseases.

## INTRODUCTION

Juvenile myelomonocytic leukemia (JMML) is an aggressive overproduction of cells in the myeloid lineage, affecting young children with a median age of 2 years, and is characterized by hypersensitivity to the cytokine granulocyte macrophage colony-stimulating factor (GM-CSF). Currently, allogeneic stem cell transplant remains the only curative treatment, with a 5-year event-free survival of only 50% [1, 2]. When studying gain-of-function (GOF) mutations in Shp2, which is the most common mutation found in JMML patients, we have previously focused on the downstream target phosphoinositide 3 kinase (PI3K). We found that genetic and pharmacologic inhibition of the hematopoietic-specific catalytic subunit of PI3K, p110δ, is uniquely important in promoting GOF Shp2-induced leukemia [3]. The genetic inhibition of p110δ *in vivo* led to reduced splenomegaly, decreased phosphorylation of Akt and Erk, and decreased progenitor cell hypersensitivity to GM-CSF in a *Shp2*^*D61Y/+*^;Mx1cre^+^ mouse model of JMML. Furthermore, pharmacologic inhibition of p110δ *in vitro* decreased phospho-Akt and -Erk and proliferation of *Shp2*^*D61Y/+*^ cells, as well as the GM-CSF hypersensitivity of mononuclear cells from primary JMML patient samples [3].

The PI3K p110δ inhibitor idelalisib was FDA-approved in 2014 for patients with relapsed chronic lymphocytic leukemia and follicular lymphoma [4]. However, the effectiveness of p110δ inhibition in JMML, a disease that lacks effective chemotherapies, has not been studied. Therefore, following our promising results demonstrating reduced GM-CSF hypersensitivity and proliferation of GOF Shp2-expressing murine cells and primary JMML cells *in vitro*, we assessed the effect of PI3K p110δ inhibition on GOF Shp2-expressing mice *in vivo* as the next step in exploring p110δ inhibition as a potential treatment strategy for JMML. We utilized the PI3K p110δ inhibitor GS-9820 (hereafter PI3Kδ inhibitor), which has superior pharmacokinetics in murine models (Dr. Stacey Tannheimer, personal communication) [5].

## RESULTS

We treated *Shp2*^*E76K/+*^;LysMcre^+^ mice between 12 and 20 weeks of age with 30mg/kg of PI3Kδ inhibitor or vehicle (0.5% w/v methylcellulose + 0.1% v/v Tween 80) BID *via* oral gavage for 21 days. This mouse model has previously been shown to develop a myeloid expansion that closely mimics human JMML and has an ideal treatment window of 12-20 weeks between disease development and death [6]. We used two separate cohorts of mice: the first cohort had seven mice per treatment group and was euthanized 16 hours following the final dose of PI3Kδ inhibitor; the second cohort had 15 mice per treatment group and was followed long-term for overall survival.

**Figure 1:**
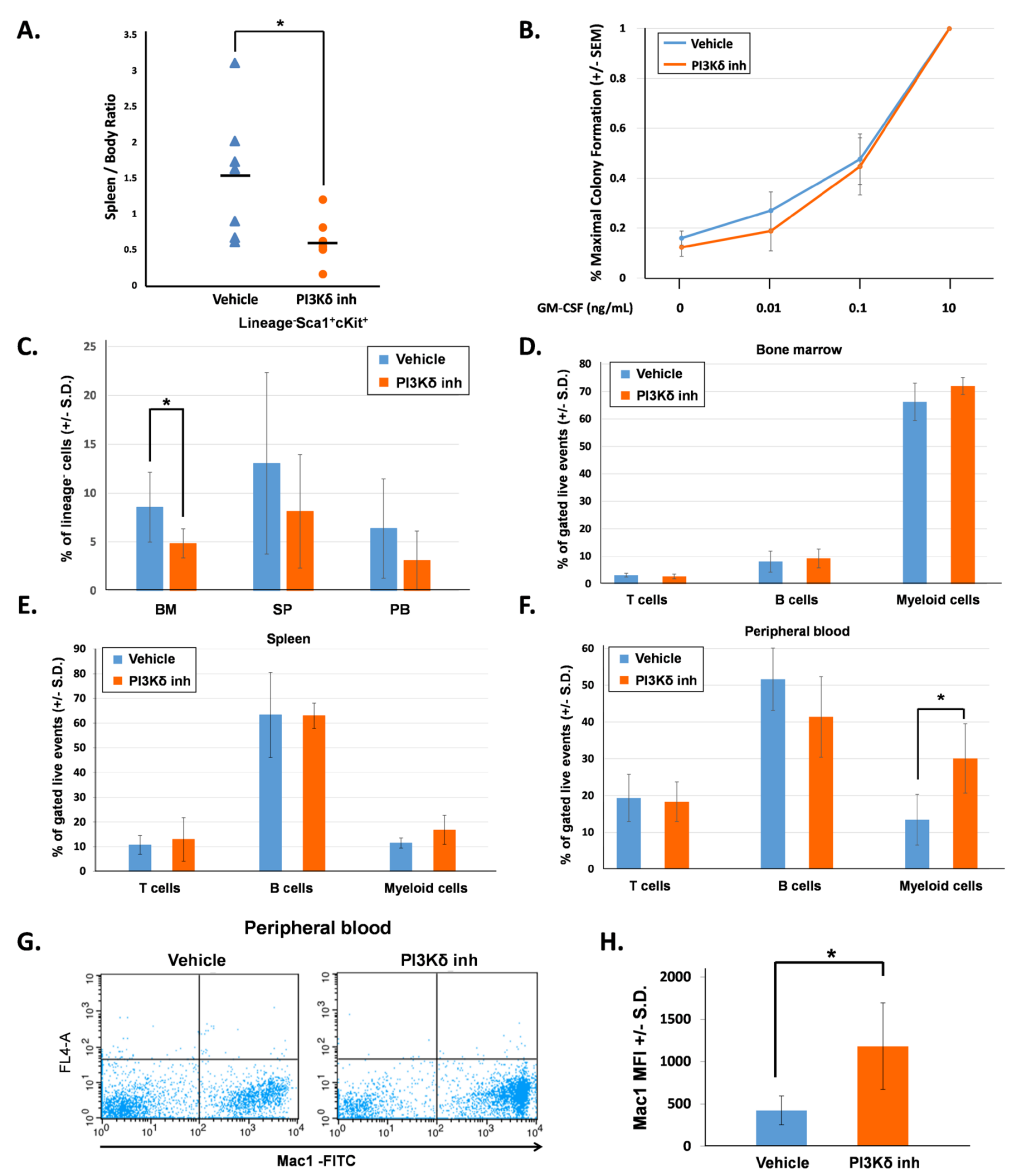
PI3K p110δ inhibition *in vivo* decreases splenomegaly and promotes myeloid cell maturation **A.** The spleen to body weight ratio of mice at the end of 21 days of treatment, *n* = 7 per group, *p = 0.03 comparing PI3Kδ inhibitor-treated mice to vehicle-treated mice, statistical analyses performed by unpaired, two-tailed, Student’s *t*-test. **B.** Number of colonies formed in methylcellulose colony forming assays, shown as a percentage of total colonies at maximum GM-CSF concentration. Bone marrow LDMNCs were collected and plated in triplicate per each concentration of GM-CSF. Colonies were counted 7 days after plating. **C.** Average percentage of Lin-Sca1^+^Kit^+^ (LSK) cells in bone marrow (BM), spleen (SP), and peripheral blood (PB), *n* = 7 per group, *p = 0.026 comparing LSK cells in the bone marrow of PI3Kδ inhibitor-treated mice to vehicle-treated mice, statistical analyses performed by unpaired, two-tailed, Student’s *t*-test. **D.** Average percentage of bone marrow T cells (CD4^+^, CD8^+^), B cells (B220^+^), and myeloid cells (Gr1^+^, Mac1^+^) gated on live events, *n* = 7 per treatment group. **E.** Average percentage of spleen T cells (CD4^+^, CD8^+^), B cells (B220^+^), and myeloid cells (Gr1^+^, Mac1^+^) gated on live events, *n* = 7 per treatment group. **F.** Average percentage of peripheral blood T cells (CD4^+^, CD8^+^), B cells (B220^+^), and myeloid cells (Gr1^+^, Mac1^+^) gated on live events, *n* = 7 per treatment group, *p = 0.05 comparing the percentage of myeloid cells in the peripheral blood of PI3Kδ inhibitor-treated mice to vehicle-treated mice, statistical analyses performed by unpaired, two-tailed, Student’s *t*-test. **G.** Representative flow cytometry diagrams showing Mac1 brightness in the peripheral blood of vehicle-treated and PI3Kδ inhibitor-treated mice. **H.** Average Mac1 mean fluorescent intensity (MFI) in peripheral blood, *n* = 7 per group, *p = 0.0029 comparing PI3Kδ inhibitor-treated to vehicle-treated mice, statistical analyses performed by unpaired, two-tailed, Student’s *t*-test.

First, we evaluated mice directly after completing 21 days of treatment (16 hours following final vehicle or PI3Kδ inhibitor dose). The PI3Kδ inhibitor-treated mice had significantly reduced spleen-to-body weight ratio in comparison to the vehicle-treated mice (Figure 1A). We measured the colony-forming ability of bone marrow low-density mononuclear cells (LDMNCs) in response to increasing concentrations of GM-CSF. 100,000 LDMNCs from each mouse were plated in semi-solid methylcellulose media containing 0, 0.01, 0.1, or 10ng/mL GM-CSF and colonies were counted 7 days later. LDMNCs isolated from PI3Kδ inhibitor-treated mice demonstrated similar GM-CSF hypersensitivity compared to the vehicle-treated mice, indicating that pharmacologic inhibition of PI3K p110δ does not permanently correct GM-CSF hypersensitivity of GOF Shp2-expressing cells (Figure 1B). To investigate how PI3Kδ inhibitor treatment induced the functional effect of reduced spleen size, we phenotypically analyzed the bone marrow, spleen, and peripheral blood for hematopoietic stem/progenitor cells (lineage^-^Sca1^+^cKit^+^, LSK) and for terminally differentiated hematopoietic cells. The frequency of LSK cells was significantly reduced in the bone marrow of PI3Kδ inhibitor-treated mice and trended lower in the spleen and peripheral blood (Figure 1C). When examining hematopoietic differentiation, we found no significant difference in myeloid progenitor populations (CMPs, GMPs, or MEPS, data not shown) or in the frequency of terminally differentiated CD4^+^, CD8^+^ (T cells), B220^+^ (B cells), and Gr1^+^Mac1^+^ (myeloid cells) in the bone marrow and spleen compartments (Figure 1D and 1E). However, the frequency of peripheral blood Gr1^+^Mac1^+^ cells was significantly increased in the PI3Kδ inhibitor-treated mice compared to vehicle-treated mice (Figure 1F). Furthermore, the Mac1^+^ mean fluorescence intensity (MFI) in the peripheral blood was significantly higher in PI3Kδ inhibitor-treated mice (Figure 1G and 1H). Collectively, these findings suggest that PI3K p110δ inhibition induced stem/progenitor terminal differentiation and reduced the self-renewal and hyperproliferation of immature myeloid cells.

**Figure 2:**
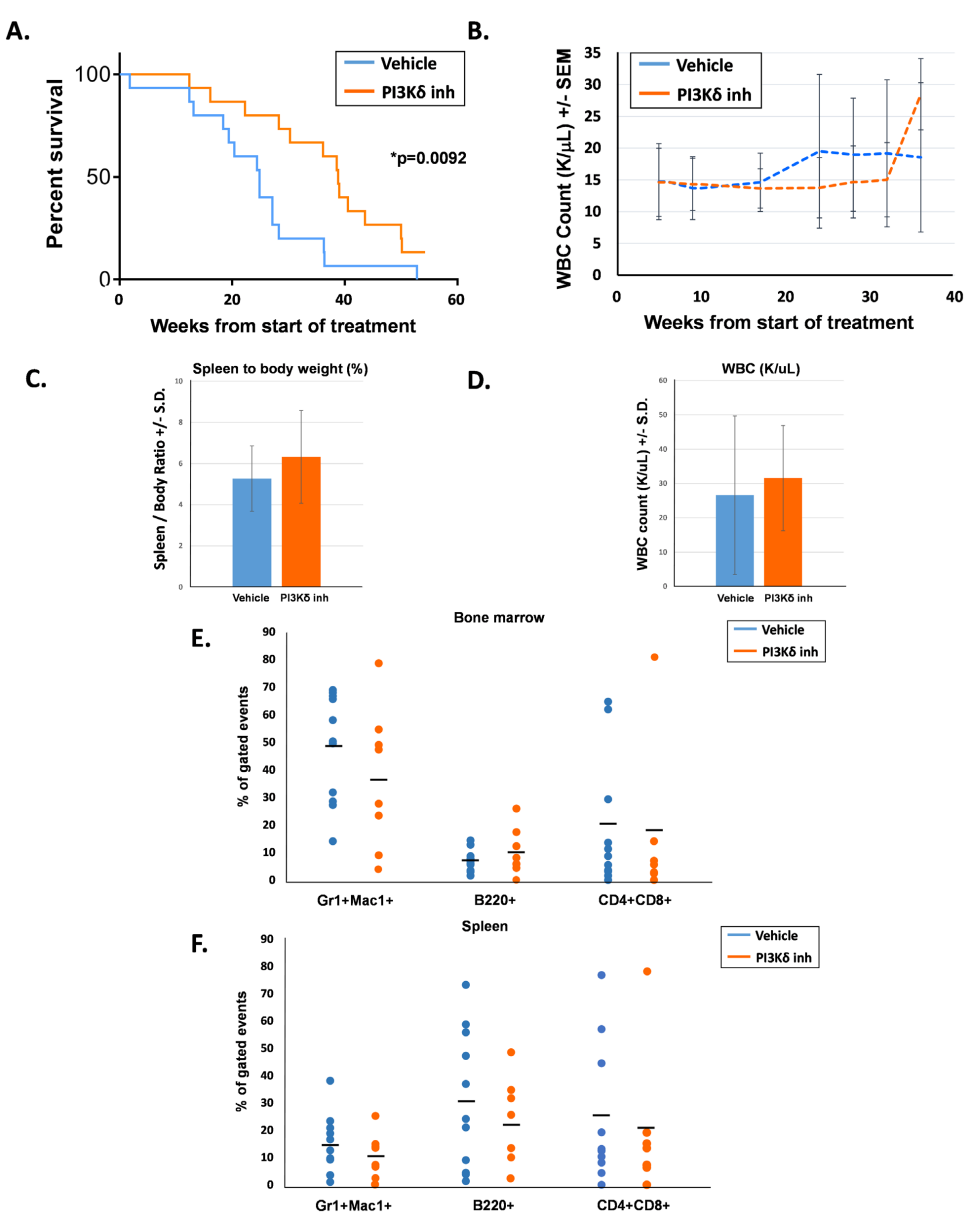
PI3Kδ inhibitor-treated mice had significantly prolonged survival compared to vehicle-treated mice **A.** Kaplan-Meier survival curve of days elapsed from start of treatment until death, *n* = 15 per treatment group, *p = 0.0092 comparing survival after treatment for PI3Kδ inhibitor-treated mice and vehicle-treated mice, statistical analyses performed by log-rank (Mantel-Cox) test. **B.** Average WBC count (K/uL) in peripheral blood of mice. **C.** Average spleen to body weight percentage at the time of death. **D.** Average WBC count (K/uL) in peripheral blood at the time of death. **E.** Percentage of bone marrow T cells (CD4^+^, CD8^+^), B cells (B220^+^), and myeloid cells (Gr1^+^, Mac1^+^) at the time of death, gated on live events. **F.** Percentage of spleen T cells (CD4^+^, CD8^+^), B cells (B220^+^), and myeloid cells (Gr1^+^, Mac1^+^) at the time of death, gated on live events.

The second cohort of 15 mice per treatment group was followed for overall survival with peripheral blood counts assessed every 4 - 6 weeks. Each treatment group consisted of 6 male mice and 9 female mice, and groups were age-matched (average age was 114 days for the vehicle-treated mice and 109 days for the PI3Kδ inhibitor-treated mice). Survival of the PI3Kδ inhibitor-treated mice was significantly prolonged compared to their vehicle-treated counterparts (Figure 2A). Serial peripheral blood WBC counts were similar between the two groups until approximately 18 weeks following the start of treatment, when WBC counts in the vehicle-treated animals started increasing compared to the PI3Kδ inhibitor-treated mice (Figure 2B). Notably, the period of time of increasing WBC counts in the vehicle-treated animals coincided with the period of time when the survival curves started to separate (20 - 35 weeks). Later at 37 weeks, the drug-treated mice also demonstrated an increase in WBC counts, which corresponded to this group’s cluster of deaths between 38 - 40 weeks.

Whenever possible, moribund mice were euthanized for analysis to assess the cause of death. Although the PI3Kδ inhibitor-treated animals demonstrated prolonged survival, at the time of euthanasia, these animals demonstrated similar levels of splenomegaly and elevated peripheral leukocyte counts compared to the vehicle-treated animals (Figure 2C and 2D). Furthermore, composition of the bone marrow and spleen in moribund animals was not significantly different between the two groups (Figure 2E and 2F). Most animals in both groups succumbed to a myeloid disease; however, both groups did demonstrate the emergence of T cell leukemia in a small number of mice, indicated by elevated levels of CD4^+^;CD8^+^ cells in the bone marrow and spleen. Taken together, these findings suggest that time-limited PI3Kδ inhibition delays mortality due to Shp2-induced leukemia, but does not alter the ultimate course of disease. These findings imply that PI3Kδ activity resumes after termination of treatment and that continued inhibition may be needed for optimal treatment of disease.

## DISCUSSION

We previously found that genetic and pharmacologic inhibition of PI3K p110δ *in vitro* normalizes hypersensitivity to GM-CSF and reduces GM-CSF-stimulated hyperproliferation of GOF Shp2-expressing murine and human cells [3]. Herein, we now demonstrate that *in vivo* inhibition of PI3K p110δ increases the survival of GOF Shp2-expressing mice. One notable observation was increased peripheral blood terminally differentiated myeloid cells (Gr1^+^Mac1^+^) in the PI3Kδ inhibitor-treated animals (Figure 1F). One potential explanation for this observation is the recently-appreciated role of PI3K p110δ in regulatory T cell function with the resulting disruption of the protective tumor cell microenvironment and peripheral distribution of tumor cells upon PI3K p110δ inhibition [7]. In the context of this JMML model, it is possible that PI3K p110δ inhibition leads to redistribution and increased differentiation of mutant myeloid cells.

In both the vehicle- and PI3Kδ inhibitor-treated animals, we observed increased peripheral blood WBC counts that temporally preceded a precipitous drop in survival (Figures 2A and 2B). The WBC spike in the PI3Kδ inhibitor-treated animals was more pronounced than that observed in the vehicle-treated group (Figure 2B). One possibility accounting for this difference is that we simply “missed” a similarly high spike in the vehicle-treated mice, which might have occurred between the 18 week and 24 week scheduled blood draws (Figure 2B). An alternative explanation is that treatment with the PI3K p110δ inhibitor allowed animals to tolerate higher circulating WBC counts before succumbing to disease.

As the armamentarium of molecularly targeted therapies continues to grow, potential application to difficult-to-treat diseases such as JMML is a valuable priority. Previous work conducted in the Braun lab demonstrated that treatment of GOF Kras- and GOF Nras-expressing mice with the pan-PI3K inhibitor, GDC-0941, or the allosteric Akt inhibitor, MK-2206, effectively ameliorated hyperactive Ras-induced disease [8]. The effectiveness of single pathway inhibition is likely limited, as drug resistance to PI3K/Akt inhibition can develop due to aberrant upregulation of the Ras-Erk pathway. However, an analysis of clinical trials found that patients receiving Ras/MEK/Erk pathway inhibitors combined with pan-PI3K inhibitors commonly experienced toxic adverse effects [9]. The differential and potentially more limited side effect profile of PI3Kδ inhibition compared to pan-PI3K inhibition [7, 10] may provide a therapeutic window to simultaneously target both the Ras-Erk and PI3K-Akt pathways in the treatment of JMML. There is a clear need for improved chemotherapeutic treatments for JMML, as the current treatment options are limited with only variable efficacy. This work supports the rationale of using PI3Kδ inhibitors in the treatment of patients with JMML and possibly other myeloid cell malignancies.

## MATERIALS AND METHODS

### Preparation of PI3Kδ inhibitor

A solution of 0.5% w/v methylcellulose (Sigma) and 0.1% v/v Tween-80 (Sigma) was made in water. Using this solution, a stock of 7.5mg/mL of GS-9820 (provided by Gilead Sciences, Inc.) was made, aliquoted into 1mL aliquots, and stored at 4°C during the 21 days of treatment. Prior to each dosing, the necessary amount of drug and vehicle were brought to room temperature and mixed thoroughly. Mice were dosed 30mg/kg PO BID.

### Animal husbandry

Mice with a conditional mutant Shp2 allele, E76K, have been previously described [6]. Mice were housed and bred in accordance with the Institutional Animal Care and Use Committee of the Indiana University School of Medicine. Expression of the E76K mutation and LysMcre were confirmed by genotyping.

### Colony forming assays

Ficoll-purified LDMNCs from bone marrow and spleen of mice were plated into methylcellulose-based assays with increasing concentrations of GM-CSF as previously described [11].

### Flow cytometry/MFI

Cell suspensions were incubated for 5 minutes with 10% rat serum (MP Biomedicals) and 0.2% BSA (Roche) in PBS, then stained for 30 minutes at 4°C with biotinylated lineage markers (Mac1, Gr1, CD4, CD8, B220, Ter119, IL7Rα, CD19, and CD3 with streptavidin PerCPcy5.5 as secondary), anti-Sca1-PE, anti-cKit-APC, anti-Gr1-FITC, anti-Mac1-APC, biotinylated anti-CD4 and anti-CD8 (with streptavidin APC as secondary), and/or anti-B220-PE (eBioscience and BD Biosciences). The analyzer used for flow cytometry was the BD LSR II and data was analyzed using CellQuest.

### Complete blood counts

Peripheral blood was collected from the saphenous vein of mice and complete blood counts were measured using a Hemavet 950 (Drew Scientific Group).

### Statistical calculations

GraphPad was used to perform the unpaired, two-tailed, Student’s *t*-tests and log-rank test.

## Abbreviations

JMML: juvenile myelomonocytic leukemia
GM-CSF: granulocyte macrophage colony-stimulating factor
GOF: gain-of-function
PI3K: phosphatidyl inositide-3 kinase
LDMNC: low-density mononuclear cell
